# Detecting Collagen Molecules at Picogram Level through Electric Field-Induced Accumulation

**DOI:** 10.3390/s20123567

**Published:** 2020-06-24

**Authors:** Romina Rega, Martina Mugnano, Emilia Oleandro, Volodymyr Tkachenko, Danila del Giudice, Gianluca Bagnato, Pietro Ferraro, Simonetta Grilli, Sebastiano Gangemi

**Affiliations:** 1 Department of Physical Science and Technology of Matter, Institute of Applied Sciences and Intelligent Systems (ISASI), National Research Council of Italy (CNR), 80078 Pozzuoli (NA), Italy; m.mugnano@isasi.cnr.it (M.M.); e.oleandro@isasi.cnr.it (E.O.); v.tkachenko@isasi.cnr.it (V.T.); d.delgiudice@isasi.cnr.it (D.d.G.); p.ferraro@isasi.cnr.it (P.F.); s.grilli@isasi.cnr.it (S.G.); 2 Department of Mathematics and Physics, University of Campania, 81100 Caserta, Italy; 3 Division of Pneumology, Papardo Hospital, Contrada Papardo, 98122 Messina, Italy; gianlucabagnato@aopapardo.it; 4 School and Operative Unit of Allergy and Clinical Immunology, Department of Clinical and Experimental Medicine, University of Messina, 98122 Messina, Italy; gangemis@unime.it

**Keywords:** collagen, lithium niobate, pyroelectric effect, pyro-electrohydrodynamic jet

## Abstract

The demand for sensors capable of measuring low-abundant collagen in human fluids has highly increased in recent years. Indeed, collagen is expected to be a biomarker for chronic diseases and could monitor their progression. Here we show detection of highly diluted samples of collagen at picogram level thanks to an innovative pyro-electrohydrodynamic jet (p-jet) system. Through the intense electric fields generated by the pyroelectric effect in a ferroelectric crystal, the collagen solution was concentrated on a small area of a slide that was appropriately functionalized to bind proteins. The collagen molecules were labeled by an appropriate fluorophore to show how the number of tiny droplets influences the limit of detection of the technique. The results show that the p-jet is extremely promising for overcoming the current detection limits of collagen-based products in human fluids, performing 10 times better than the enzyme-linked immunosorbent assay (ELISA) and thus paving the way for the early diagnosis of related chronic diseases.

## 1. Introduction

Collagens are the most abundant proteins in mammals. Although the same collagen type may have isoforms with different molecular structures, they all have in common a structural motif represented by the triple helix with three alpha chains [1]. The collagen superfamily comprises 28 members [2] and they constitute important components of connective tissue, playing a crucial role in its development and regeneration [3]. Collagen synthesis is highly regulated by multiple intracellular and extracellular steps. Abnormal collagen modification and/or degradation occur in chronic and other pathological conditions [4,5,6], which can generate potential circulating biomarkers in human fluids, useful for an early diagnosis. This occurs for example in case of idiopathic pulmonary fibrosis (IPF) [7], cancer [8], osteoporosis [9], arthritis [10] and fibrosis [11]. Anyway, the exact nature and evolution of collagen damage are still poorly understood [12]. 

Nowadays, many assays exist to measure the products of collagen metabolism in the serum, plasma and urine [13,14,15], and they are used as biomarkers for chronic diseases to monitor their progression noninvasively [16,17,18]. Another procedure, based on a graphene oxide-based fluorescence resonance energy transfer (FRET) sensor [19], is able to detect unstructured collagen fragments, whereby degradation by proteolytic enzymes plays a critical role in many biological processes. Moreover, Desogere et al. have developed an approach based on direct molecular imaging, functionalizing 16–amino acid disulfide-bridged cyclic peptide [7]. Conversely, Zitnay et al. use the collagen hybridizing peptide (CHP) labeled with carboxyfluorescein (CF) to track down and localize this biomarker [20].

All these techniques are very difficult to employ in clinical laboratories. Indeed, the ordinary method to quantify markers in body fluid is the enzyme-linked immunosorbent assay (ELISA), a powerful tool for quantifying proteins and qualifying their state of activation in complex biological samples [21,22]. Despite being specific, simple and rapid, the ELISA presents important limitations, such as limited sensitivity, a fundamental parameter in the early diagnosis. The current limit of detection (LOD) of the Human Pro-Collagen I alpha 1, with ELISA assay is 31.2 pg/mL [23] and new methods are desirable in order to overcome such a limit. Higher sensitivity of collagen biomarker in peripheral fluid would provide new perspectives for an early diagnosis of diseases characterized by circulating collagen fragments. Indeed, collagen biomarker levels in plasma have been associated with survival rate in patients affected by advanced pancreatic cancer [24], pulmonary arterial hypertension (PAH) [25], chronic obstructive pulmonary disease (COPD) [26] and glioblastoma, the most common and aggressive primary central nervous system tumor [27,28].

Here we show how an innovative system that we call the “pyro-electrohydrodynamic jet” (p-jet) is able to accumulate collagen molecules, thus improving the LOD down to less than 3 pg/mL, namely 10-fold better than ELISA kits are capable to measure. The technique exploits the pyroelectric effect generated by polar dielectric crystals such as lithium niobate (LN) under an electrode- and nozzle-free configuration. The electric field generated by the pyroelectric effect acts electro-hydrodynamically on the sample of liquid in which the biomolecules are dispersed, allowing the deposition of small liquid volumes and thus by guiding and accumulating biomolecules directly onto a restricted area of a functionalized deposition slide. Thanks to this approach it is possible to obtain an increase in sensitivity since it avoids the diffusion phenomenon that generally occurs in conventional methods. We introduced the p-jet system recently to demonstrate the proof-of-concept of accumulating low abundant biomolecules by using test samples of oligonucleotides and gliadin, and next to detect low abundant molecules of lactose [29,30]. Here we demonstrate the applicability of the technique to a real test case where collagen samples mimic the low abundant collagen peptides in peripheral body fluids. The reliability of the technique is demonstrated in case of fluorescent-labeled collagen molecules diluted serially in PBS. Results are compared for two different configurations of the p-jet system in order to compare and optimize the performance in terms of accumulation efficiency, by changing the source of the pyroelectric effect stimulation.

## 2. Materials and Methods

### 2.1. Target Slides

We used SuperAmine 2 microarray substrates (ArrayIt^®^) for binding the collagen molecules. They consist of microarray substrates with positively charged reactive amino groups on a polished, atomically flat glass substrate. This product is ideal for researchers and microarray manufacturing organizations engaged in protein, oligonucleotide and cDNA microarray printing. The substrates are polished to high precision to achieve atomic-level smoothness of ±20 angstroms and present an amino group density of about 5 × 10^12^ per mm^2^.

### 2.2. Lithium Niobate and Pyroelectric Effect

Lithium niobate (LN) is a rhombohedral crystal belonging to the point group 3 m. Thanks to its unique optical, electronic and physical properties, LN has a wide range of applications such as nonlinear optical devices, compact pyroelectric sources of x-rays, piezoelectric resonance biosensors to name but a few. The only obstacle in the way of its growing industrial use expensive high-quality mechanical elaboration of LN samples is currently reduced due to implementation of micromachining CNC (Computer Numerically Controlled) [31] and laser micromachining [32] techniques. 

The LN crystals were bought from Crystal Technology Inc. in the form of both sides being polished 500 μm thick c-cut wafers with a diameter of 3 inches. The wafer were cut into square samples 2 × 2 cm^2^ in size by a standard diamond saw. The spontaneous polarization *P_s_* changes according to Δ*P_i_* α *p_i_*Δ*T*, where *P_i_* is the coefficient of the polarization vector, *p_i_* is the pyroelectric coefficient and Δ*T* is the temperature variation (*p_i_* = −8.3 × 10^−5^ C m^−2^ °C^−1^ for LN at 25 °C). At equilibrium, when the thermal stimulation is turned off, the spontaneous polarization of the LN crystal is completely screened by the external screening charge and no electric field is present [33,34,35]. When the crystal is stimulated through a temperature variation, a surface charge density α = *p_i_* Δ*T* appears locally due to uncompensated charges and a high electric field (E_NL_ ~10^7^ V/m) is generated on the surface of the LN crystal. Recently, we demonstrated for the first time the possibility of using the pyroelectric effect for a wide variety of applications, ranging from biological to soft matter manipulation application [36,37,38,39,40].

The pyro-electric field applied to a liquid drop leads to redistribution of the mobile charges in the liquid and, when sufficiently strong, deforms the liquid into a conical tip from which thin liquid jets are released (nature of pyro-electrohydrodynamic jetting is explained in detail in the “Results” section). 

### 2.3. CO_2_ Laser

In the laser-based configuration we used a CO_2_ laserthat emits at 10.6 microns (wavelength that falls within the absorption band of the LN crystals) with an output power of 10 W. The stimulation is impulsive for about 200 ms. Since the impulse is not ultra-short the IR electromagnetic radiation, when it hits the crystal surface, generates vibrational states associated with the local heating of the crystal by approximately 35 °C, thus enabling the generation of pyroelectric surface charges density on the crystal about 10^−2^ C/m^2^ [41] and an estimated electric potential up to 5 × 10^3^ V. The CO_2_ laser output was modulated by a conventional 5 V transistor-transistor logic (TTL) and was equipped with a collinear He-Ne laser beam for precise alignment. The focal point had a diameter of about 4 mm.

### 2.4. Micro-Heater

In the micro-heater-based configuration the heating system was obtained by integrating a titanium coil on the lower side of the LN crystal, so a circulating current produces strictly local heating due to the Joule effect. The resistive coil, in a meander shape, was fabricated by using standard optical photolithography and thin film deposition via dc sputtering in a high vacuum system (basic pressure of 1.2·10^−5^ Pa). A subsequent lift-off process defined the final geometry, resulting in a meander coil having a thickness of 250 nm and covering an area of (3.5 × 3.5) mm^2^. The thermal stimulus is controlled at microscale by means of power dissipation driven by a traditional voltage generator. The estimated electric potential reached 2 × 10^3^ V for temperature variation of 15 °C. We note that above 75 °C the crystal developed cracks initially and finally broke due to the thermal expansion of the titanium coil [33].

### 2.5. Collagen Samples

We used collagen from human placenta Bornstein and Traub type I (cat. no. C7774, Sigma-Aldrich) was used for the experiment. Collagen powder was carefully dissolved in 0.5 M acetic acid at 1 mg/mL at 37 °C with magnetic stirring for 1 day, to assist dissolution. They were labelled according to the following procedure. Molecular probe Alexa Fluor 647 Protein Labeling Kit (cat. no. A20173) was used to label collagen Type I. In order to adjust the pH of the protein solution, 50 μL of 1 M sodium bicarbonate buffer and 45 μL of sodium hydroxide (NaOH) 5 M was add to 0.5 mL of protein to raise the final pH of the reaction mixture to 7.5–8.3. Afterwards labelling reaction mixture was prepared according to guidelines reported in the datasheet. The reaction mixture was loaded onto a purification column (available with the kit), containing a gel filtration resin in phosphate-buffered saline (pH 7.2), and after, the labelled protein was collected in a tube, while the band of unreacted dye was retained in the void volume. The concentration of labelled collagen was calculated measuring the adsorbance at 280 nm and subtracting the adsorbance at 650 nm correct by a 0.03 factor, to account for absorption of the dye at 280 nm. Afterwards, different serial dilutions in phosphate-buffered saline (PBS) were prepared 500 ng/mL, 300, 60, 30, 15 and 3 pg/mL, respectively. 

### 2.6. Fluorescence Scanner

A conventional fluorescence scanner InnoScan 710 (Innopsys, Carbonne, France) was used for reading the target slides. Main scan parameters: Laser source at 635 nm and 10 mW power; pixel size 3 μm and scan speed of 10 lines s-1; detection gain 25% photomultiplier (PMT).

## 3. Results

**Figure 1:**
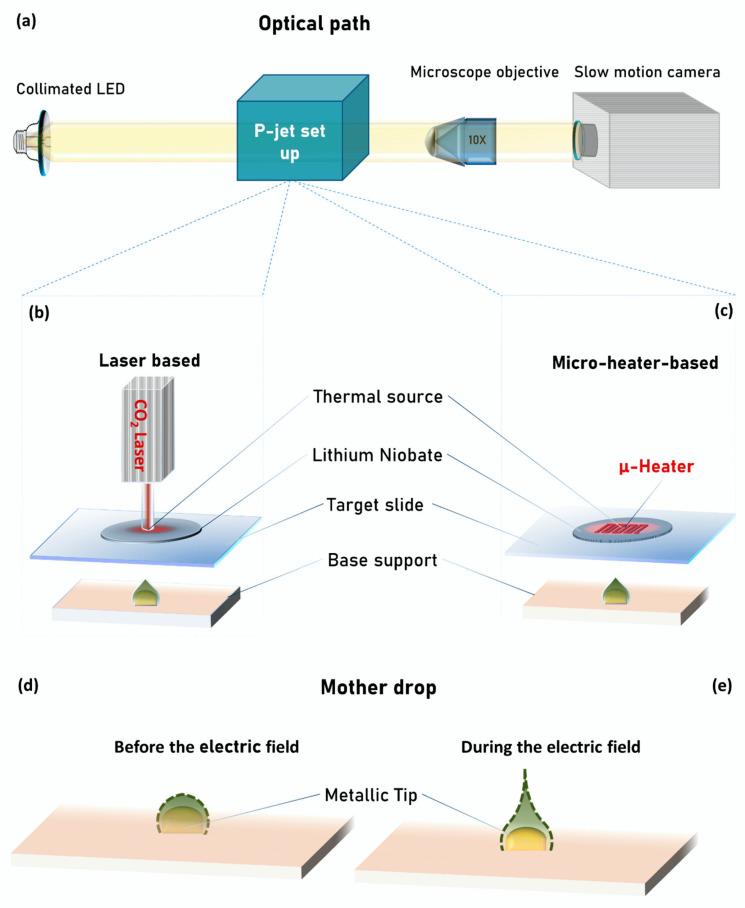
Schematic view of: (**a**) pyro-electrohydrodynamic jet (p-jet) set-up optical path; (**b**) p-jet system in laser-based configuration; (**c**) p-jet system in micro-heater-based configuration; shape of the mother drop (**d**) before and (**e**) during the electric field application.

Figure 1a shows the schematic view of the optical path we used here for the side view inspection of the jetting process. 

A collimated LED illuminates the liquid jets laterally, while a microscope objective (10×) magnifies the images of the jets and projects them onto a slow-motion camera (Motion Pro Y3-S1, IDT Corporation, Newark, NJ, USA, pixel size of 10.85 × 10.85 μm) linked to a laptop for recording the movie of the jets (see more details in [42,43,44]). 

Figure 1b,c shows schematically the p-jet system developed here for concentrating collagen molecules in low abundant solutions. It is made basically of four components: (1) Base support; (2) target slide; (3) LN crystal; and (4) thermal source. Each of the first three components is equipped with three-axes translation stages for both horizontal and vertical positioning with precision on a micrometric scale. The base support is made of a round shaped tip (height 0.2 mm and width 0.9 mm) firmly inserted into a rubber layer (around 1 mm thick) for stability reasons, while the target slide is a commercial SuperAmine slab able to immobilize proteins with high efficiency (see Methods for details). The LN crystal is also commercial and is used here for exploiting its ability to exhibit the pyroelectric effect (see Methods for details). Concerning the thermal source, we used here two different configurations that we call “laser-based” (see Figure 1a) and “micro-heater-based” (see Figure 1b). The first one uses a CO_2_ laser (Universal Laser Systems Inc., Scottsdale, AZ, USA, see Methods for details) for heating locally the LN crystal, while the second one uses a titanium coil fabricated on the surface of the crystal (see Methods for details). In case of the laser-based configuration, the CO_2_ laser head is placed vertically at about tens of centimeters far from the LN crystal, while in case of the micro-heater-based configuration, the heater is integrated directly on the back face of the LN crystal. In both cases the thermal source is aligned with the position of the round shaped tip on the base support. The temperature of the two thermal stimulation systems is constantly controlled through a standard infrared camera. 

**Figure 2:**
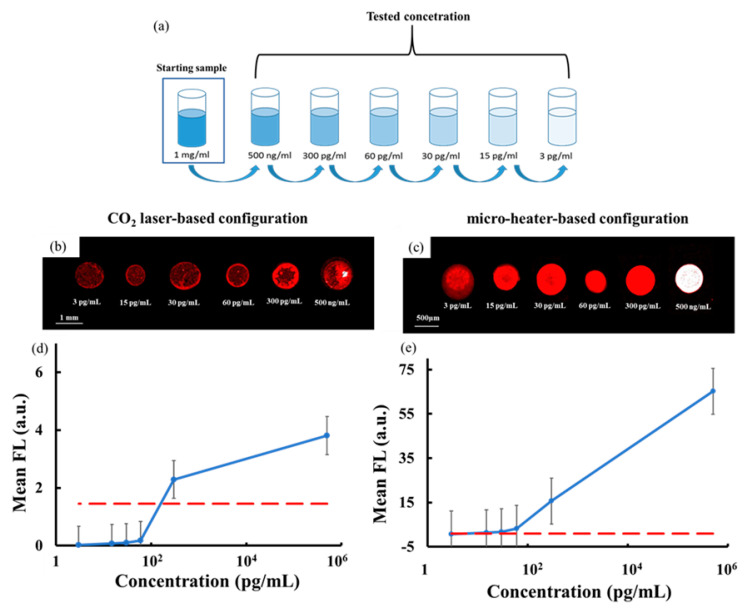
(**a**) Schematic view of the nr.6 tested diluted solutions of collagen obtained, starting from the mother solution at 1 mg/mL in PBS. Typical scanner image of the collagen spots: (**b**) In case of the CO_2_ laser-based configuration; (**c**) in case of the micro-heater-based configuration. Plot of mean fluorescence signal as a function of the solution concentration in case of (**d**) the CO_2_ laser-based configuration and of (**e**) the micro-heater-based configuration. The dashed red line corresponds to the three standard deviations of the background intensity.

We prepared six samples of highly diluted collagen solution, with the serial dilutions obtained starting from the mother solution at 1 mg/mL in PBS, as shown in Figure 2a.

The samples were labelled by standard conjugation with AlexaFluor647 (Thermo Fisher Scientific, Waltham, MA, USA, see Methods for details about the collagen solutions). We chose this range of dilutions in order to compare the detection performance of our system with current techniques, which have a limit of detection (LOD) around 30 pg/mL [23]. It is noteworthy that the collagen solution samples were not mixed with any mixing buffer in order to prevent, as best as possible, interference with the immobilization chemistry on the SuperAmine slide. 

For each collagen solution we proceeded in the following way: We deposited manually 0.2 μL of the collagen solution onto the tip of the base support by using a Hamilton syringe. The use of the tip as support allows us to expose a thinner liquid meniscus to the electric field, thus guaranteeing high repeatable droplet ejection [44,45]. We call this drop the “mother drop” and Figure 1d shows the schematic side view, where the dashed line corresponds to the liquid–air interface. The distance between the mother drop and the target slide is approximately 800 μm, which represents the activation distance for the jetting event [46]). The LN crystal is heated locally by the absorbed radiation of the CO_2_ laser in case of the laser-based configuration and by the Joule effect in case of the micro-heater-based one. The thermal stimulation generates a non-negligible surface density charge through the pyroelectric effect (see Methods for details) [33]. As a consequence, an electric field is originated in correspondence of the heated spot and propagates though the target slide up to the meniscus of the mother drop. Analogously to what happens in other applications that we presented recently [30,47,48,49], the meniscus of the mother drop charges under the action of the electric field. As a consequence, a repulsive Coulomb force arises between these charges of the same sign and deforms the meniscus into the so-called Taylor cone [48,49,50], from which apex liquid jets are ejected (see Figure 1e). The magnitude of the electric field influences the number of jets, the volume of the single dispensed drop and the activation distance of the jetting event as described in [51]. In fact, the mother drop dispenses liquid periodically till the electric field is active and the separation distance between the base support and target slide is less than the activation distance. In the laser-based configuration, at the same activation distance of micro-heater-based configuration, the electric field that invades the mother drop is higher and; therefore, a greater volume of liquid is dispensed in each drop, but the higher temperature to which the mother drop is subject causes its fast evaporation, indeed reducing the number of dispensed jets (see results for more details).

In order to ensure the repeatability of the jetting event in both configurations, the temperature gradient reached in each heating cycle is always the same (ΔT = 35 °C in the laser-based configuration and ΔT = 15 °C in the micro-heater-based configuration). After each heating pulse, the necessary time (tens of seconds) is waited for reaching a new equilibrium state of the LN at room temperature.

Starting from the mother drop, it is possible to obtain a sequence of different jets that are all deposited directly on a limited area of the target slide, thus achieving what we call “accumulation” of molecules. This allows us to confine the collagen molecules in a microscale immobilization area, thus increasing significantly the fluorescence (FL) signal-to-noise ratio. We performed this accumulation for each of the six diluted samples under both configurations and we read the target slide by a conventional FL scanner Innoscan 710 (see Materials and Methods for details) to measure the FL signals at the 635 nm wavelength channel. Three replicates of the experiments were performed for each sample and Figure 2b,c shows a typical scanner image of the spots in case of laser-based and micro-heater-based configurations, respectively.

The FL spots had a round shape with a homogeneous distribution of the FL signal and a typical diameter of 600 µm in case of the laser and 500 µm in case of the micro-heater. The FL spots were analyzed by the commercial software MAPIX (version 5.5.0, Innopsys, Carbonne, France) and Figure 2d,e shows the behavior of the FL signal for each collagen concentration averaged over the values obtained by the three replicates of the experiments. It is noteworthy that the FL signal for each spot corresponds to the mean spot pixel intensity with the background subtracted at the referenced wavelength of 635 nm. The red dashed line corresponds to the limit of detection calculated as three times the standard deviation of the background intensity. These results show clearly that while the laser-based configuration provides a LOD value of 300 pg/mL, the micro-heater-based configuration allows us to achieve a LOD value of 3 pg/mL, one order of magnitude better than that reported in [23]. 

**Figure 3:**
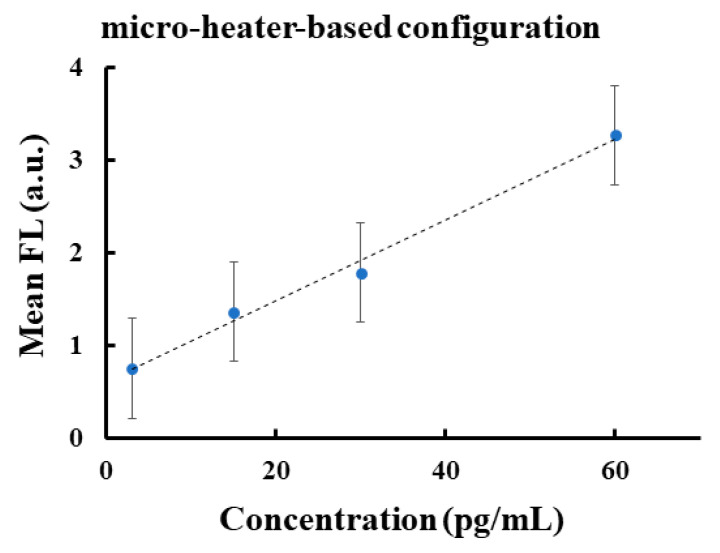
Plot of regression of linear range of mean fluorescence as a function of the concentration in the case of the micro-heater-based configuration.

Therefore, we focused our attention on the micro-heater-based configuration by evaluating the corresponding calibration curve. Figure 3 shows the linear regression that best fits the linearity range between 3 and 60 pg/mL of the FL signal for the micro-heater configuration showing the R^2^ value close to 1. 

The FL spots corresponding to the laser-based configuration (see Figure 2b) were produced by the accumulation of three repeatable jets of about 60 nL each. In this case the temperature on the LN crystal changed from the room value up to about 60 °C, thus causing the rapid evaporation of the collagen medium and the potential denaturation of the protein [52]. Moreover, this reduced the volume of the mother drop during the jetting events and; therefore, the amount of accumulating jets. In case of the micro-heater configuration, the electric field was generated simply by switching on the voltage supply in order to exploit the rising temperature of the titanium coil circuit and; therefore, the Joule effect, which brings the crystal temperature from room value up to about 40 °C in approximately 60 s, with a maximum power dissipation of 350 mW. The accumulation in this case was achieved through four repeatable jets of about 30 nL each, able to produce spots with a high signal-to-noise ratio down to 3 pg/mL (see Figure 2c). The results in Figure 2 demonstrate that the intensity values of the FL spot obtained by the micro-heater is about 20 times higher than those obtained by the laser for concentrations up to 60 pg/mL. We attribute this result to the additional jet obtained by using the micro-heater configuration. 

## 4. Conclusions

We showed that p-jet technology has a sensitivity reporting the detection down to 3 pg/mL; therefore, one order of magnitude higher when compared to ELISA-based assays. Successful accumulation of collagen molecules from a mother drop of about 0.2 μL directly onto the surface of the target slide was achieved. The resulting FL spots exhibited high signal-to-noise ratio and provided a linearity range from 3 to 60 pg/mL with a LOD value of 3 pg/mL, thus well below the current limits in the field. This innovative LOD improvement was achievable thanks to the ability of p-jet to overcome the diffusion limits typically encountered in ELISA-based assays, by dispensing tiny jets that concentrate the collagen molecules onto an accumulation site of the target slide. Therefore, the molecules were spatially confined into a very small area, thereby increasing the signal intensity per unit area. Moreover, in this study we also reported comparative analysis between two different modalities for stimulating pyroelectric effect in p-jet, namely by micro-heater and laser, respectively. Results show that the micro-heater configuration performed much better. In fact, the micro-heater was able to control the heating process at the microscale allowing us to achieve temperature values around 40 °C, thus reducing the evaporation rate of the mother drop compared to the laser-based configuration. This made the number of accumulating jets increase significantly. In perspective, this system will allow clinicians to detect low abundant collagen-related products in human fluids in order to achieve an early and non-invasive diagnosis of fatal diseases such as IPF, systemic sclerosis, PAH or glioblastoma.
